# Chronic Lymphocytic Leukaemia: Census of Patients Treated in Italian Haematology Units

**DOI:** 10.4084/MJHID.2015.056

**Published:** 2015-11-01

**Authors:** Gianfranco Salvi, Idanna Innocenti, Francesco Autore, Luca Laurenti

**Affiliations:** 1 Research Director of Kantar Health Italy; 2 Haematology Department, Policlinico Gemelli, Università Cattolica del sacro Cuore – Rome

## Abstract

This study was conducted by contacting the population of the Italian haematology units and collecting from 68% of them data concerning the number of patients with chronic lymphocytic leukaemia visited over the previous 12 months, with the aim of obtaining an overview of the treatment of this disease and comparing the results with the prevalence estimates found in literature.

The projection obtained (about 17,000 patients visited in the previous 12 months) is probably overestimated because of double-counting of patients who may have been treated at two different facilities during the year, although it is also underestimated since the internal medicine units were not involved. The balance of these two opposite factors is not known.

It is important to bear in mind the approximation with which the count was performed in facilities for which no official data were available.

Albeit with these limits, the results obtained are in line with some existing prevalence data and make it possible to determine the portion of patients at different Binet stages and in the various age ranges, identifying the corresponding therapeutic treatments. Use of the CIRS scale to classify patients as FIT and UNFIT was seen to be still somewhat limited.

## Introduction

Chronic Lymphocytic Leukaemia (CLL) is the most common form of leukaemia in the Western world, with an incidence that in Italy is estimated at between 5.0 and 5.5 cases per 100,000 men and between 3.5 and 4.0 cases every 100,000 women;^1^,^2^,^4^ only the populations of Australia, United States and Ireland are characterised by a higher incidence.^7^,^9^,^10^ The risk of a diagnosis of CLL increases significantly with age: the estimated incidence for the population over 70 years of age is approximately 20 cases every 100,000.^3^,^5^

The prevalence data available for Italy, calculated on the basis of mean survival, are discordant: some sources talk about 20,000–22,000 cases,^3^,^8^ others about approximately 12,000 cases.^6^

The idea of performing a census of patients with CLL originates, on the one hand, from the need to estimate the number of individuals with the disease using an alternative approach and, on the other, from the need to collect information of use for understanding the way this disease is treated in the different parts of our country.

The census is in itself an ambitious aim that by definition has to tackle the many difficulties implicit to data collection. The method proposed, which is based on the quantification of the number of patients visited in each facility, represents a compromise that does not aim to provide exact results, rather to offer realistic estimates of the phenomena that may influence the choice of therapy concerning the treatment of CLL in Italy. Since the survey was conducted in almost 70% of the facilities defined originally, the authors believe that the data obtained and corresponding projections, albeit with the limits that will be discussed, constitute a very useful contribution to improving current knowledge in this area.

## Materials and Methods

The data was collected using a questionnaire containing questions of interest (Annex 1). These questions concerned the overall numbers and percentages of patients visited/treated, rather than specific patients. In short, the questionnaire included the following items:

- The characteristics of the hospital facility
- The number of specialists working in the unit
- The number of specialists in the unit treating CLL
- The number of patients with CLL visited in the unit over the previous 12 months
- The number of patients with CLL visited in the unit over the previous month
- The percentage of patients diagnosed over the previous 12 months
- The percentages of patients in the various Binet stages A, B and C
- The mean visit frequency for the different Binet stages
- The percentages of patients in the different age ranges
- The percentages of patients from other regions (migration)
- The percentages of patients in the different treatment lines
- The distribution of the treatments administered over the different treatment lines outside clinical studies
- The therapeutic objectives in the different treatment lines
- Use of the CIRS scale and calculation of the score obtained

The choice of the population of facilities to be contacted focused on Haematology units (initial list of 204 centres), excluding internal medicine and general medicine facilities. This choice, which was due primarily to the limited concentration of patients with CLL in these units, most likely makes the final projections an underestimate.

In order to collect reliable data concerning the whole unit, it was essential to identify an appropriate contact person to be interviewed. Consequently, the initial screening of the questionnaire included a specific question asking whether the respondent was able to provide data for the facility as a whole. In addition, for the haematology units with a presumably higher number of patients, the authors used a list of specialists who are known to be a reference point for the treatment of CLL in their units.

The information on the number of patients with CLL visited at the facility was sought in two different ways: for the previous month and for the previous 12 months, specifying which of the two values should be considered more reliable and whether this value was based on the data available or on a personal estimate. This made it possible to correct the values provided according to the following criteria, in order to obtain a better estimate of the number of patients visited over the previous 12 months:

**Table:** 

CASE A (17%)	More reliable value: previous 12 months	Source: Data available	Decision: Value provided
CASE B (25%)	More reliable value: previous 12 months	Source: Personal estimate	Decision: Case-by-case
CASE C (11%)	More reliable value: previous month	Source: Data available	Decision: Correct value
CASE D (47%)	More reliable value: previous month	Source: Personal estimate	Decision: Correct value

The facilities that provided effectively available data (cases A+C) rather than personal estimates were a minority; however, these facilities are concentrated amongst those treating a higher number of patients (on average, 111 patients over the previous 12 months vs. 79 in cases B+D).

The correction was performed using an algorithm that, starting from the number of patients visited in the previous month (considered more simple to quantify in the absence of other information), projects the annual value taking into consideration the mean frequency of patient visits in each of the Binet stages (Annex 2).

It is important to point out that the correction, in most cases, did not significantly influence the 12-month estimates declared during the interview and that in all produced a 7% reduction in patients.

The B cases were examined individually because they were more uncertain; when values provided and corrected values were discordant the centres were re-contacted and verification interviews performed with another contact person. More generally, the same approach was taken for structures that had provided inconsistent or unrealistic values. Overall, a double-check was performed on approximately 30% of facilities.

The interview was conducted by telephone and only in a few cases in person and always subject to appointment; during the first contact the respondent was told about the aims of the study, emphasising the need to collect reliable data on the treatment of CLL at the facility. The interviews were conducted by specialised personnel with consolidated experience in the medical field. The survey lasted approximately 4 months, from November 2014 to February 2015.

The final projections for the universe of haematology units were performed by considering the size of the 68 centres that refused the interview by dividing the facilities into four ranges according to the number of beds.

## Results

### Estimate of the number of patients

Overall, in 136 out of a total of 204 facilities identified (of which 5 did not administer treatment), the number of patients with CLL visited in the previous 12 months was 11,526 units; if this number is projected over the entire population of haematology units, we obtain an estimate of 17,044 patients (28 cases every 100,000 inhabitants).

In order to evaluate this value, it is necessary to consider the possibility that the same patient may, during the year, have been treated at more than one centre. From this point of view the information collected concerning healthcare mobility can be of help: on average, 15% of patients visited in a facility lives in a different region to that in which the unit is found. These patients are more likely to be visited also in a centre in their home region.

The projection of the diagnoses of CLL made in the previous 12 months was 2,966 patients (4.9 cases every 100,000 inhabitants), equal to approximately 17% of the whole. Newly diagnosed patients are particularly prone to double-counting, as the diagnosing facility in many cases is different to that in which the patient is subsequently treated. We therefore believe that the effective percentage and number of new diagnoses is in actual fact significantly lower.

At hub centres, which see a higher number of patients, the percentage of new diagnoses was higher.

The units contacted that treat CLL, employee an average of 8–9 specialists and residents (median =7), of whom approximately half deal actively with the condition (median = 3).

### Binet stages

**Figure 1:**
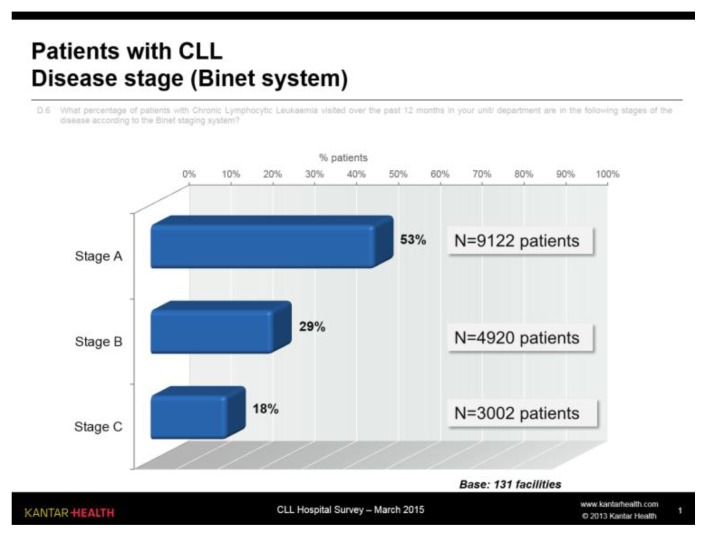


The specialists interviewed were asked to break down the patients visited over the previous year at the facility according to Binet stage: in all (figure 1), approximately half (53%) of the patients were in stage A (seen on average every 5–6 months), 29% were in stage B (seen on average every 3–4 months) and 18% were in stage C (seen on average every 1–2 months).

It is likely that, compared to the total population of patients with CLL, the weight of patients in stage A is underestimated because these patients, apart from the diagnosis phase, are seen even less frequently than once a year, in which case they were not counted.

For the same reason, the visit frequency of patients in stage A is probably overestimated compared to the total number of patients in stage A.

### Age ranges

**Figure 2:**
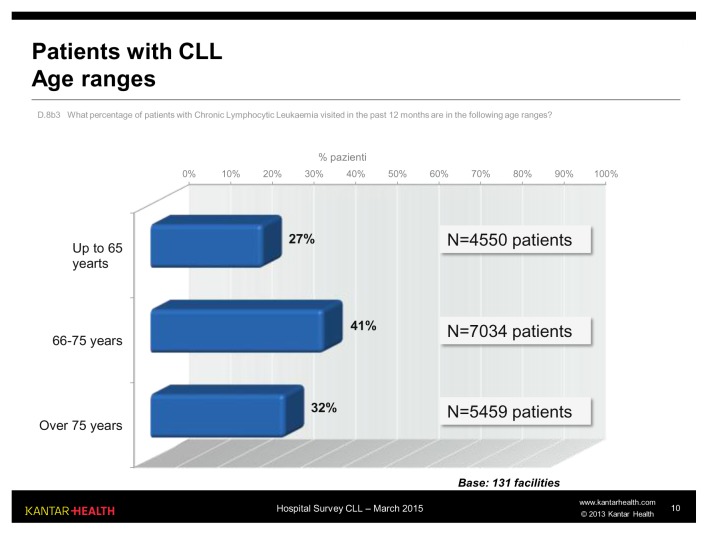


Age is one of the most influential variables in patient evaluation and the choice of treatment: of the three age ranges (up to 65 years, 66–75 years and over 75 years) used in the questionnaire, the most common was the intermediate range (41%, approximately 7000 patients). The patients who for the treatment of CLL are usually defined “young” (up to 65 years) account for approximately one quarter (figure 2).

### Test

**Figure 3:**
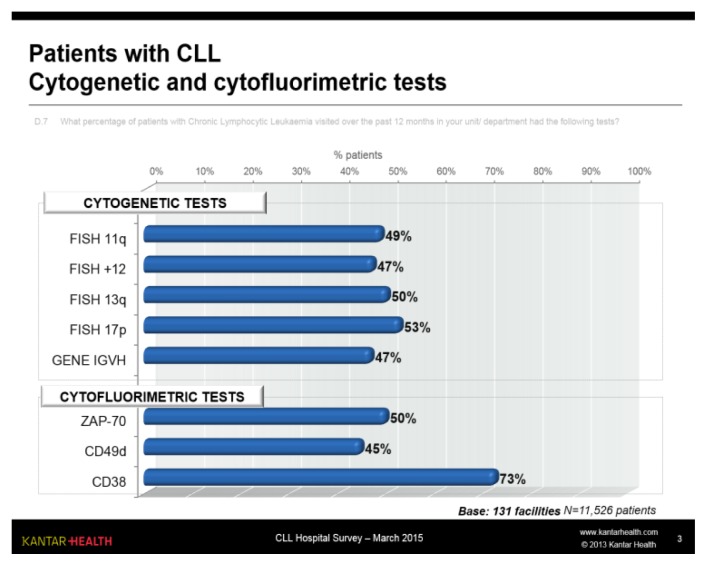


It was estimated that approximately half of the patients visited over the previous 12 months had each of the different cytogenetic tests (figure 3), with a slightly higher frequency only for 17p deletion, which is more selective for therapy. GENE IGVH, with FISH +12, was the cytogenetic test less frequently performed.

Of the various flow cytometry tests, the conventional CD38 test was used, whereas use of the CD49d test was still somewhat limited.

### Treatment lines

**Figure 4:**
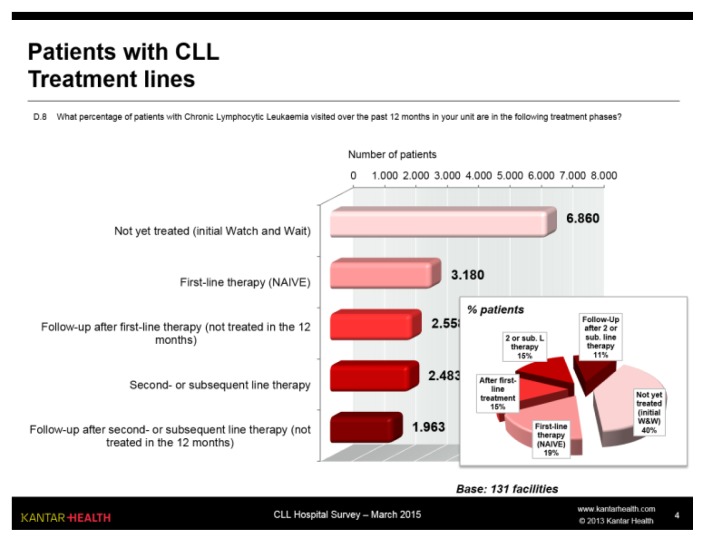


40% of patients with CLL seen during the previous 12 months was awaiting initial treatment (Watch & Weight); if we also exclude patients in follow-up after first- and second-line treatment, we see that approximately one third of patients was on treatment: in all approximately 5,500 patients, just over half of whom on first-line treatment (figure 4).

The doctors interviewed estimated that, on average, approximately 20% of W&W patients will start treatment in the next 12 months.

### Treatments administered

We then collected the distributions of the pharmacological treatment administered in the unit to patients visited in the previous year, making a distinction between the three different patient age ranges: up to 65 years, 66–75 years, over 75 years.

#### First-line therapy

**Figure 5:**
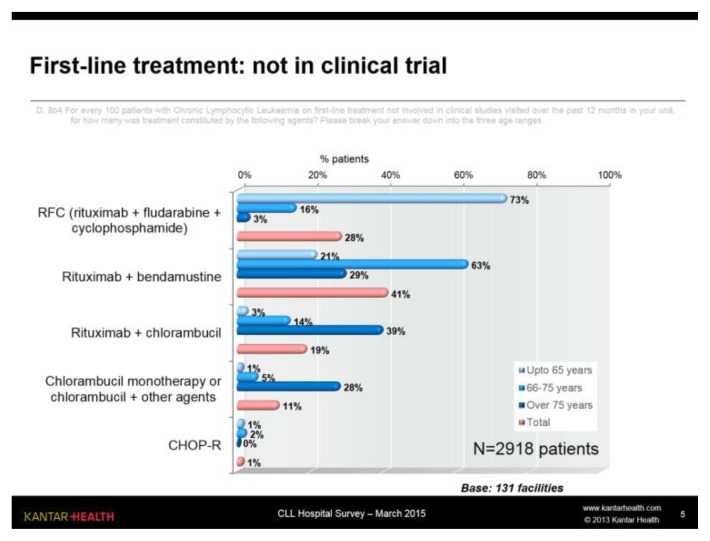


By excluding the 8% of patients involved in clinical studies, the treatments administered as first-line therapy (figure 5) are greatly concentrated on RFC (Rituximab - Fludarabine - Cyclophosphamide) in young patients (73%) and on R-Benda (Rituximab - Bendamustine) in patients aged between 66 and 75 years (63%). For older patients, with poorer general conditions, treatments with Chlorambucil (in combination with Rituximab, but also in monotherapy and in combination with other drugs) are very common; the percentage of patients treated with R-Benda drops in this case to 29% (as shown in the rituximab + bendamustine chart in figure 5).

The therapeutic objective of first-line treatment is, with a similar frequency (41%), increase in OS or increase in PFS / CR, whereas symptom control/palliative care is already defined as a treatment objective in almost 20% of cases.

#### Second- and subsequent line therapy

**Figure 6:**
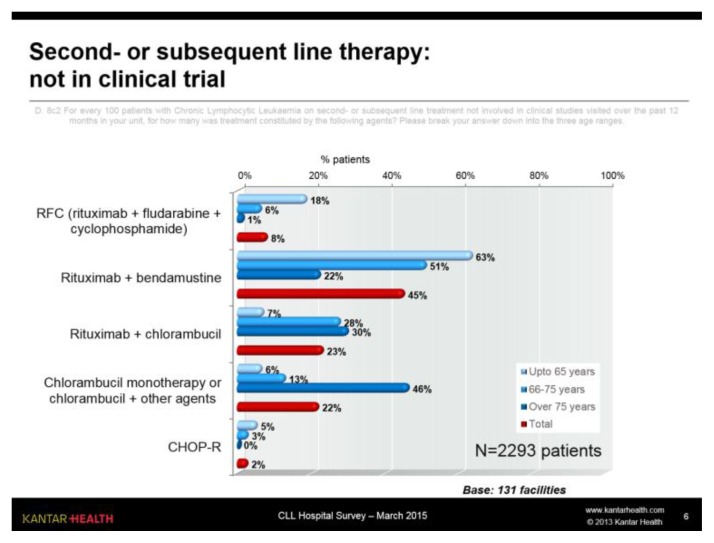


Again excluding the 8% of patients involved in clinical studies, in treatment lines subsequent to the first (figure 6) the most commonly used treatment overall is R-Benda, especially in patients up to 75 years.

Chlorambucil is very commonly used in patients over 65, in combination with Rituximab or often in other regimens in elderly patients.

**Figure 7:**
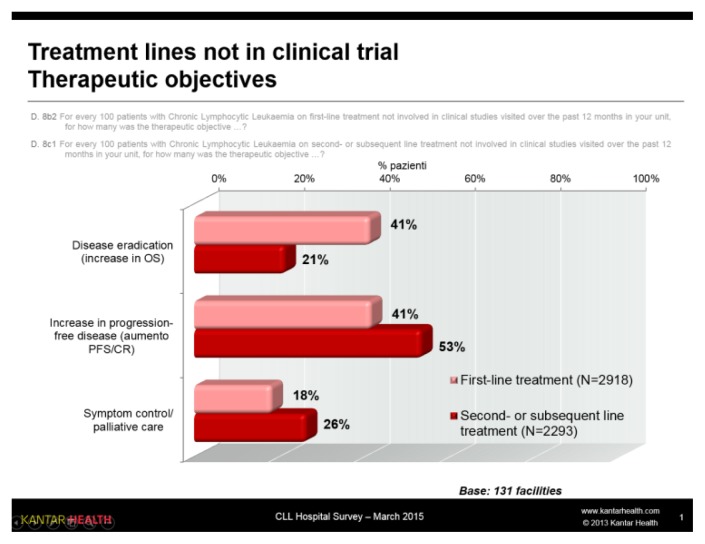


In these treatment lines (figure 7), the most common therapeutic objective is an increase in PFS/ CR (53% of cases), which is higher than in first-line treatment (26%). As expected, OS is the primary endpoint for a mere 21% of patients. In this subset of patients, the category of patients for whom symptom control/ palliative care is the therapeutic objective is obviously higher (26%) than was reported for patients receiving first-line therapy.

### Cirs Scale and Score Calculation

**Figure 8:**
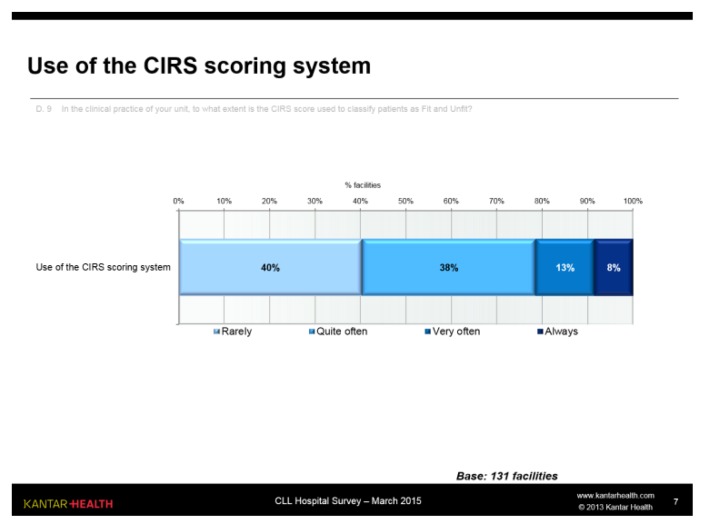


The CIRS score classifying patients as FIT and UNFIT (where a score of ≤ 6 is classified as fit and > 6 as unfit) is partially used in the clinical practice of the units: 40% of respondents replied that this happens rarely, another 38% replied “quite often”. Only a minority, primarily in the centres treating more patients, make frequent (13%) or continuous (8%) use of the scoring system (figure 8).

This result may have a close relationship with the choice of treatment, as all the most recent treatment indications refer to the CIRS score.

## Discussion and Conclusion

The vast coverage obtained amongst haematology facilities allows us to make comparisons with existing epidemiological data on CLL. Considering that the estimates obtained from this study present two limits of an opposite nature, the double counting of certain patients (overestimation) and the exclusion of medical units (underestimation), the number of patients obtained was seen to be in line with the incidence and prevalence of the disease most commonly reported in literature for this country.

The data obtained is in agreement with that found in literature in terms of disease stage, age, lines of therapy and treatment options. However, the data concerning the biological tests used are surprising. Currently, the most important parameter, also in the light of the novel treatments available, is the study of 17p/p53 deletion, followed by 11q mutation and testing. CD38 and Zap-70 tests would now appear to be rather obsolete, given their unclear clinical value, whereas CD49d has shown a greater prognostic value. These data reflect the methodology and technology used, highlighting that in some centres flow cytometry (which is now essential for diagnosing CLL) is more commonly used, whilst FISH and PCR testing is less common. The same can be said for CIRS score calculation: it is probably used more by centres conducting a greater number of clinical trials or with a higher number of patients. It is essential for use of the CIRS scale to become part of clinical practice precisely in the light of the new medicinal products now available.

This study is the first large-scale survey of its kind to be conducted in Italy and provides us with incidence data and a picture of the clinical and biological characteristics of patients with CLL. In addition, its added value is the possibility to obtain an evaluation that reflects today’s therapeutic orientation and allows us to hypothesise how the current treatment scenario will change with the advent of new medicinal products.

## Annex 2: CORRECTION ALGORITHM

To use a formula, if:

NPM = Number of patients visited in the past month
ND = % of patients diagnosed in the past 12 months
PA = % of patients in Binet stage A
PB = % of patients in Binet stage B
PC = % of patients in Binet stage C
VA = Visit frequency in Binet stage A (in months)
VB = Visit frequency in Binet stage B (in months)
VC = Visit frequency in Binet stage C (in months)
NPY = Estimate of the number of patients visited in the past year

the algorithm is expressed as:

NPY = NPM * 6 * ND * (1/6 + 5/6 * (PA * VA / 6 + PB * VB / 6 + PC * VC / 6)) + NPM * 12 * (1-ND) * (PA * VA / 12 + PB * VB / 12 + PC * VC / 12)

## Annex 3: FACILITIES INVOLVED IN THE STUDY

**Table:**

HOSPITAL	CITY	PROV	REGION
AON SS. ANTONIO E BIAGIO E CESARE ARRIGO	ALESSANDRIA	AL	PIEMONTE
OSP CARDINALE GUGLIELMO MASSAIA	ASTI	AT	PIEMONTE
AZIENDA SANITARIA OSPEDALIERA SANTA CROCE E CARLE	CUNEO	CN	PIEMONTE
OSPEDALE MAGGIORE SS. ANNUNZIATA	SAVIGLIANO	CN	PIEMONTE
CLINICA UNIVERSITARIA	TORINO	TO	PIEMONTE
AO ORDINE MAURIZIANO UMBERTO I	TORINO	TO	PIEMONTE
ASLTO4 OSPEDALE EX MAURIZIANO	LANZO TORINESE	TO	PIEMONTE
OSPEDALE SANT’ANDREA	VERCELLI	VC	PIEMONTE
OSPEDALE CIVILE S. ANNA	S. FERMO DELLA BATTAGLIA	CO	LOMBARDIA
OSPEDALE VALDUCE	COMO	CO	LOMBARDIA
ISTITUTI OSPITALIERI DI CREMONA	CREMONA	CR	LOMBARDIA
OSPEDALE ALESSANDRO MANZONI	LECCO	LC	LOMBARDIA
AZIENDA OSPEDALIERA SAN GERARDO	MONZA	MB	LOMBARDIA
OSPEDALE DI CIRCOLO	DESIO	MB	LOMBARDIA
OSPEDALE NIGUARDA CA’ GRANDA	MILANO	MI	LOMBARDIA
ISTITUTO CLINICO HUMANITAS	ROZZANO	MI	LOMBARDIA
OSPEDALE SAN RAFFAELE	MILANO	MI	LOMBARDIA
OSPEDALE LUIGI SACCO AZIENDA OSPEDALIERA POLO UNIVERSITARIO	MILANO	MI	LOMBARDIA
OSPEDALE SAN CARLO BORROMEO	MILANO	MI	LOMBARDIA
OSPEDALE CIVILE G. FORNAROLI	MAGENTA	MI	LOMBARDIA
A. O. GUIDO SALVINI - OSPEDALE DI CIRCOLO - MONUMENTO AI CADUTI PER LA PATRIA	RHO	MI	LOMBARDIA
ISTITUTO NAZIONALE DEI TUMORI-FONDAZIONE IRCCS	MILANO	MI	LOMBARDIA
ISTITUTO EUROPEO DI ONCOLOGIA	MILANO	MI	LOMBARDIA
AZIENDA OSPEDALIERA CARLO POMA	MANTOVA	MN	LOMBARDIA
IRCCS POLICLINICO SAN MATTEO	PAVIA	PV	LOMBARDIA
OSPEDALE EUGENIO MORELLI DI SONDALO	SONDALO	SO	LOMBARDIA
OSPEDALE DI CIRCOLO E FONDAZIONE MACCHI	VARESE	VA	LOMBARDIA
OSPEDALE DI CIRCOLO DI BUSTO ARSIZIO	BUSTO ARSIZIO	VA	LOMBARDIA
OSPEDALE SANT ANTONIO ABATE	GALLARATE	VA	LOMBARDIA
POLICLINICO UNIVERSITARIO DI PADOVA	PADOVA	PD	VENETO
OSPEDALE SANTA MARIA MISERICORDIA	ROVIGO	RO	VENETO
PO CA’ FONCELLO	TREVISO	TV	VENETO
PRESIDIO OSPEDALIERO VITTORIO VENETO	VITTORIO VENETO	TV	VENETO
PRESIDIO OSPEDALIERO DI MIRANO - ULSS 13	MIRANO	VE	VENETO
OSPEDALE ALTO VICENTINO	SANTORSO	VI	VENETO
OSPEDALE SAN BASSIANO DI BASSANO DEL GRAPPA	BASSANO DEL GRAPPA	VI	VENETO
AZIENDA OSPEDALIERA UNIVERSITARIA INTEGRATA - OSPEDALE POLICLINICO ‘GIAMBATTISTA ROSSI’ DI BORGO ROMA	VERONA	VR	VENETO
OSPEDALE GIROLAMO FRACASTORO	SAN BONIFACIO	VR	VENETO
IRCCS CENTRO RIFERIMENTO ONCOLOGICO	AVIANO	PN	FRIULI V. G.
OSPEDALI RIUNITI - OSPEDALE MAGGIORE	TRIESTE	TS	FRIULI V. G.
OSPEDALE SANTA MARIA DELLA MISERICORDIA DI UDINE	UDINE	UD	FRIULI V. G.
OSPEDALE S MARTINO	GENOVA	GE	LIGURIA
OSPEDALE VILLA SCASSI - SAMPIERDARENA	GENOVA	GE	LIGURIA
OSPEDALE CIVILE	SESTRI LEVANTE	GE	LIGURIA
OSPEDALE SAN MARTINO DI GENOVA	GENOVA	GE	LIGURIA
OSPEDALE CIVILE FELETTINO	LA SPEZIA	SP	LIGURIA
POLICLINICO SANT’ORSOLA-MALPIGHI	BOLOGNA	BO	EMILIA ROM.
ISTITUTO SCIENTIFICO ROMAGNOLO PER LO STUDIO E LA CURA DEI TUMORI (I.R.S.T.)	MELDOLA	FC	EMILIA ROM.
AZIENDA OSPEDALIERA UNIVERSITARIA DI FERRARA - ARCISPEDALE SANT’ANNA	FERRARA	FE	EMILIA ROM.
OSPEDALE DEL DELTA	LAGOSANTO	FE	EMILIA ROM.
AZIENDA OSPEDALIERO - UNIVERSITARIA POLICLINICO DI MODENA	MODENA	MO	EMILIA ROM.
OSPEDALE GUGLIELMO DA SALICETO	PIACENZA	PC	EMILIA ROM.
OSPEDALE SANTA MARIA DELLE CROCI	BAGNACAVALLO - RAVENNA	RA	EMILIA ROM.
OSPEDALE DEGLI INFERMI	RIMINI	RN	EMILIA ROM.
OSPEDALE SAN DONATO DI AREZZO	AREZZO	AR	TOSCANA
AOU CAREGGI	FIRENZE	FI	TOSCANA
OSPEDALE DEL MUGELLO	BORGO SAN LORENZO	FI	TOSCANA
OSPEDALE SAN GIUSEPPE	EMPOLI	FI	TOSCANA
OSPEDALE CAMPO DI MARTE	LUCCA	LU	TOSCANA
NUOVO OSPEDALE DELLA VERSILIA	LIDO DI CAMAIORE	LU	TOSCANA
OSPEDALE CIVICO	CARRARA	MS	TOSCANA
AZIENDA OSPEDALIERA PISANA SANTA CHIARA	PISA	PI	TOSCANA
OSPEDALE SS COSMA DAMIANO	PESCIA	PT	TOSCANA
AZIENDA OSPEDALIERA UNIVERSITARIA SENESE - POLICLINICO LE SCOTTE	SIENA	SI	TOSCANA
OSPEDALE GENERALE REGIONALE TORRETTE - OSPEDALI RIUNITI	ANCONA	AN	MARCHE
OSPEDALE CIVILE ENGLES PROFILI	FABRIANO	AN	MARCHE
OSPEDALE CARLO URBANI	JESI	AN	MARCHE
PRESIDIO OSPEDALIERO SENIGALLIA	SENIGALLIA	AN	MARCHE
OSPEDALE C. E G. MAZZONI	ASCOLI PICENO	AP	MARCHE
OSPEDALE CIVILE AUGUSTO MURRI	FERMO	FM	MARCHE
OSPEDALE SAN SALVATORE MURAGLIA	PESARO	PU	MARCHE
OSPEDALE CITTA’ DI CASTELLO	CITTA’ DI CASTELLO	PG	UMBRIA
OSPEDALE FABRIZIO SPAZIANI	FROSINONE	FR	LAZIO
ISTITUTO “MARCO PASQUALI” ICOT	LATINA	LT	LAZIO
OSPEDALE SANTA MARIA GORETTI	LATINA	LT	LAZIO
OSPEDALE SANT’EUGENIO	ROMA	RM	LAZIO
POLICLINICO TOR VERGATA	ROMA	RM	LAZIO
AZIENDA OSPEDALIERA SANT’ANDREA	ROMA	RM	LAZIO
ISTITUTO NAZIONALE TUMORI REGINA ELENA IRCCS	ROMA	RM	LAZIO
OSPEDALE SAN CAMILLO FORLANINI	ROMA	RM	LAZIO
AZIENDA OSPEDALIERA SAN GIOVANNI ADDOLORATA	ROMA	RM	LAZIO
PTP NUOVO REGINA MARGHERITA EX OSP NUOVO REGINA MARGHERITA	ROMA	RM	LAZIO
OSPEDALE SANDRO PERTINI	ROMA	RM	LAZIO
OSPEDALE REGINA APOSTOLORUM	ALBANO LAZIALE	RM	LAZIO
OSPEDALE SANTO SPIRITO	ROMA	RM	LAZIO
OSPEDALE SAN PIETRO FATEBENEFRATELLI	ROMA	RM	LAZIO
POLICLINICO UNIVERSITARIO AGOSTINO GEMELLI	ROMA	RO	LAZIO
POLICLINICO UMBERTO I DI ROMA	ROMA	RO	LAZIO
POLICLINICO UNIVERSITARIO CAMPUS BIO-MEDICO	ROMA	RO	LAZIO
AZIENDA SANITARIA LOCALE VITERBO - OSPEDALE BELCOLLE	VITERBO	VT	LAZIO
OSPEDALE CIVILE SS ANNUNZIATA	SULMONA	AQ	ABRUZZO
OSPEDALE SS FILIPPO E NICOLA - LOCALITA’ TRE CONCHE	AVEZZANO	AQ	ABRUZZO
OSPEDALE CIVILE GIUSEPPE MAZZINI	TERAMO	TE	ABRUZZO
OSPEDALE ANTONIO CARDARELLI	CAMPOBASSO	CB	MOLISE
FONDAZIONE DI RICERCA E CURA GIOVANNI PAOLO II DI CAMPOBASSO	CAMPOBASSO	CB	MOLISE
OSPEDALE FERDINANDO VENEZIALE	ISERNIA	IS	MOLISE
OSPEDALE SAN GIUSEPPE MOSCATI DI AVELLINO	AVELLINO	AV	CAMPANIA
PRESIDIO OSPEDALIERO GAETANO RUMMO	BENEVENTO	BN	CAMPANIA
AZIENDA OSPEDALIERA S ANNA E S SEBASTIANO	CASERTA	CE	CAMPANIA
OSP CIVILE S GIUSEPPE MOSCATI	AVERSA	CE	CAMPANIA
AZIENDA OSPEDALIERA ANTONIO CARDARELLI	NAPOLI	NA	CAMPANIA
FONDAZIONE SENATORE G. PASCALE - ISTITUTO PER LA CURA E LA RICERCA DEI TUMORI	NAPOLI	NA	CAMPANIA
SECONDA UNIVERSITA’ DEGLI STUDI DI NAPOLI	NAPOLI	NA	CAMPANIA
AOU FEDERICO II	NAPOLI	NA	CAMPANIA
OSPEDALE SAN GENNARO	NAPOLI	NA	CAMPANIA
OSPEDALE SAN GIULIANO DI GIULIANO IN CAMPANIA	GIUGLIANO DI CAMPANIA	NA	CAMPANIA
AZIENDA OSPEDALIERA UNIVERSITARIA OO.RR. SAN GIOVANNI DI DIO RUGGI D’ARAGONA	SALERNO	SA	CAMPANIA
OSPEDALE CIVILE A. TORTORA	PAGANI	SA	CAMPANIA
ISTITUTO TUMORI “GIOVANNI PAOLO II” I.R.C.C.S.	BARI	BA	PUGLIA
OSPEDALE DI VENERE	CARBONARA DI BARI	BA	PUGLIA
AZIENDA OSPEDALIERO-UNIVERSITARIA CONSORZIALE POLICLINICO DI BARI	BARI	BA	PUGLIA
OSPEDALE MONSIGNOR DIMICCOLI	BARLETTA	BT	PUGLIA
AZIENDA OSPEDALIERA UNIVERSITARIA OSPEDALE RIUNITI	FOGGIA	FG	PUGLIA
OSPEDALE GIUSEPPE MOSCATI	STATTE	TA	PUGLIA
IRCSS CROB	RIONERO IN VULTURE	PZ	BASILICATA
OSPEDALE SAN CARLO DI POTENZA	POTENZA	PZ	BASILICATA
OSPEDALE GIANNATTASIO	ROSSANO	CS	CALABRIA
OSPEDALE CIVILE FERRARI	CASTROVILLARI	CS	CALABRIA
PRESIDIO OSPEDALIERO DI SOVERATO-CHIARAVALLE	SOVERATO	CZ	CALABRIA
OSPEDALE NUOVO CONTRADA FERRANTAZZO	LAMEZIA TERME	CZ	CALABRIA
OSPEDALE CIVILE SAN GIOVANNI DI DIO	CROTONE	KR	CALABRIA
OSPEDALE BUSINCO	CAGLIARI	CA	SARDEGNA
OSPEDALE SAN MARTINO DI ORISTANO	ORISTANO	OR	SARDEGNA
OSPEDALE CIVILE SS. ANNUNZIATA DI SASSARI	SASSARI	SS	SARDEGNA
OSPEDALE FERRAROTTO ALESSI	CATANIA	CT	SICILIA
FONDAZ. IST.ONCOLOGICO MEDITERRANEO	VIAGRANDE	CT	SICILIA
OSPEDALE GRAVINA E S. PIETRO	CALTAGIRONE	CT	SICILIA
AZIENDA OSPEDALIERA UNIVERSITARIA POLICLINICO G. MARTINO	MESSINA	ME	SICILIA
AO PAPARDO PIEMONTE	MESSINA	ME	SICILIA
OSPEDALE VINCENZO CERVELLO	PALERMO	PA	SICILIA
AZIENDA OSPEDALIERA UNIVERSITARIA POLICLINICO “PAOLO GIACCONE”	PALERMO	PA	SICILIA
FONDAZIONE ISTITUTO G GIGLIO	CEFALU’	PA	SICILIA
OSPEDALE CIVICO E BENFRATELLI DI PALERMO	PALERMO	PA	SICILIA
CASA DI CURA LA MADDALENA DI PALERMO	PALERMO	PA	SICILIA
OSPEDALE CIVILE	RAGUSA	RG	SICILIA
OSPEDALE VITTORIO EMANUELE II	CASTELVETRANO	TP	SICILIA
